# Incidence of human rabies and characterization of rabies virus nucleoprotein gene in dogs in Fujian Province, Southeast China, 2002–2012

**DOI:** 10.1186/s12879-017-2698-9

**Published:** 2017-08-30

**Authors:** Jian-Ming Zhang, Zhi-Shan Zhang, Yan-Qin Deng, Shou-Li Wu, Wei Wang, Yan-Sheng Yan

**Affiliations:** 1 Clinical Laboratory, The Affiliated Quanzhou First Hospital of Fujian Medical University, No. 248 East Street, Quanzhou City, Fujian Province 362002 China; 2 School of Public Health, Fujian Medical University, Fuzhou City, Fujian Province 350004 China; 3 Fujian Provincial Center for Disease Control and Prevention, Fuzhou City, Fujian Province 350001 China; 4 Fujian Provincial Key Laboratory of Zoonosis Research, Fuzhou City, Fujian Province 350001 China; 5 Jiangsu Institute of Parasitic Diseases, No. 117 Yangxiang, Meiyuan, Wuxi City, Jiangsu Province 214064 China; 6 Key Laboratory of National Health and Family Planning Commission on Parasitic Disease Control and Prevention, Wuxi City, Jiangsu Province 214064 China; 7 Jiangsu Provincial Key Laboratory on Parasites and Vector Control Technology, Wuxi City, Jiangsu Province 214064 China

**Keywords:** Rabies, Epidemiological features, Rabies virus, Nucleoprotein gene, Fujian Province

## Abstract

**Background:**

Rabies is a global fatal infectious viral disease that is characterized by a high mortality after onset of clinical symptoms. Recently, there has been an increase in the incidence of rabies in China. The aim of this study was to investigate the incidence of human rabies and characterize the rabies virus nucleoprotein gene in dogs sampled from Fujian Province, Southeast China from 2002 to 2012.

**Methods:**

Data pertaining to human rabies cases in Fujian Province during the period from 2002 through 2012 were collected, and the epidemiological profiles were described. The saliva and brain specimens were collected from dogs in Quanzhou, Longyan and Sanming cities of the province, and the rabies virus antigen was determined in the canine saliva specimens using an ELISA assay. Rabies virus RNA was extracted from canine brain specimens, and rabies virus nucleoprotein gene was amplified using a nested RT-PCR assay, followed by sequencing and genotyping.

**Results:**

A total of 226 human rabies cases were reported in Fujian Province from 2002 to 2012, in which 197 cases were detected in three cities of Quanzhou, Longyan and Sanming. ELISA assay revealed positive rabies virus antigen in six of eight rabid dogs and 165 of 3492 seemingly healthy dogs. The full-length gene fragment of the rabies virus nucleoprotein gene was amplified from the brain specimens of seven rabid dogs and 12 seemingly healthy dogs. Sequence alignment and phylogenetic analysis revealed that these 19 rabies virus nucleoprotein genes all belonged to genotype I, and were classified into three genetic groups. Sequencing analysis showed a 99.7% to 100% intra-group and an 86.4% to 89.3% inter-group homology.

**Conclusions:**

This study is the first description pertaining to the epidemiological characteristics of human rabies cases and characterization of the rabies virus nucleoprotein gene in dogs in Fujian Province, Southeast China. Our findings may provide valuable knowledge for the development of strategies targeting the prevention and control of rabies.

## Background

Rabies is a fatal disease that is mainly transmitted by the bites of dogs. Globally, rabies is responsible for approximately 55,000 deaths each year [1]. In China, a total of 108,412 human rabies cases have been reported between 1950 and 2004, yielding to India as a country with the second highest number of rabies victims [2].

During the past decade, there has been a sharp increase in the incidence of human rabies in Fujian Province, Southeast China [3–5]. This trend reflects a rapid increase in the number of domestic dogs that lack effective canine vaccination and control measures, especially in the countryside [6]. However, the incidence of human rabies and the genotypes of dog rabies viruses remain unknown in Southeast China until now. This study aimed to investigate the incidence of human rabies and characterize dog rabies virus in Fujian province, Southeast China from 2002 to 2012.

## Methods

### Ethical statement

This study was approved by the Ethical Review Committee of Fujian Provincial Center for Disease Control and Prevention (CDC) (permission number: FJCDC-001207), and the study protocol was reviewed by the Institutional Review Board of Fujian CDC. All animal experiments were performed according to the Guidelines for the Care and Use of Laboratory Animals, and written informed consent was obtained from all human rabies cases described in this study.

### Collection of human rabies cases

The data pertaining to human rabies cases were captured from the annual reports of Fujian CDC (Fuzhou, China) during the period from 2002 through 2012, and the epidemiological profile of the cases was described.

### Canine samples

**Fig. 1:**
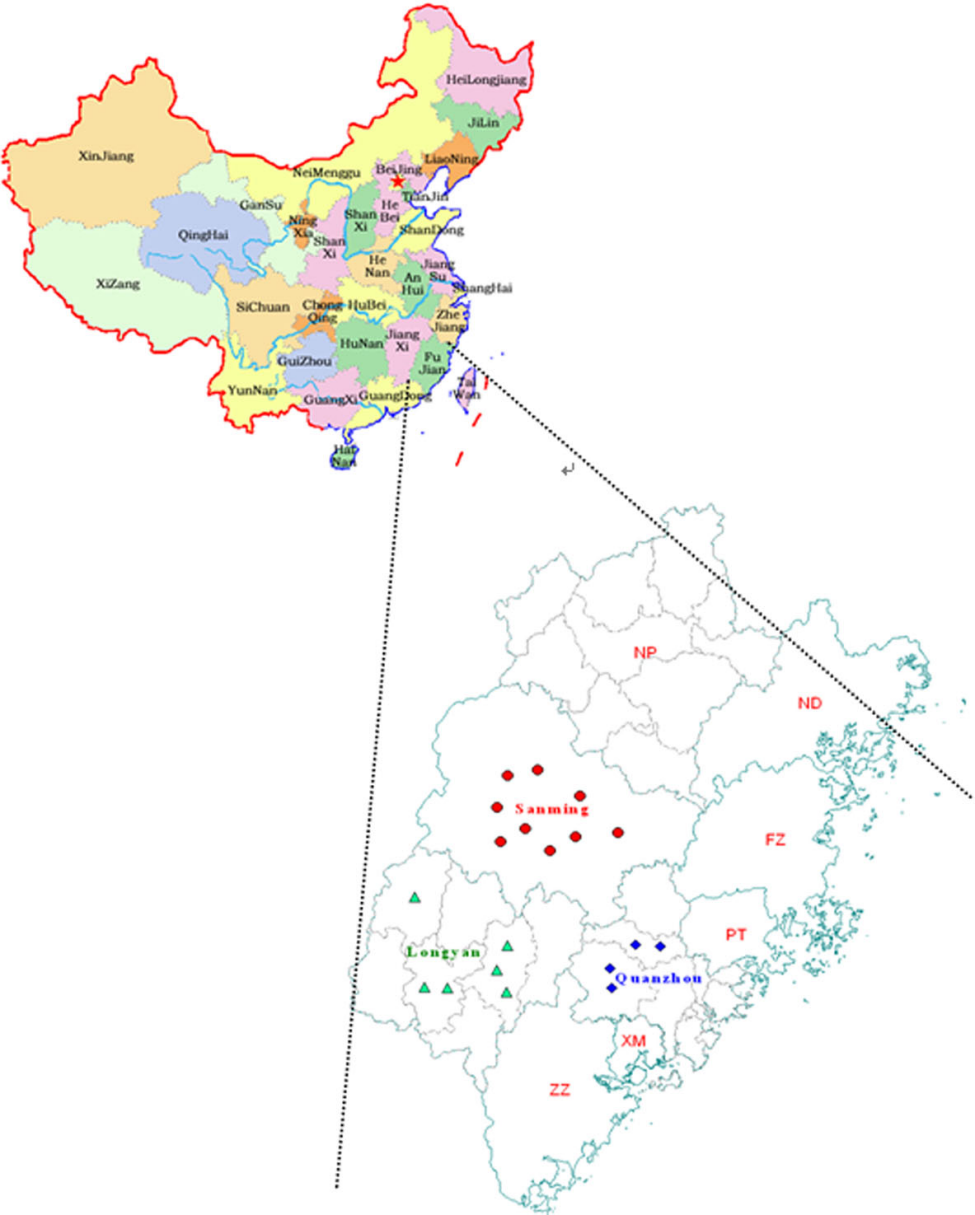
Geographical distribution of rabies virus isolates in Fujian Province, Southeast China

Dogs were sampled from Quanzhou, Longyan and Sanming cities, which had the highest incidence of human rabies in Fujian Province, Southeast China (Fig. 1). Table 1 shows the geographical origin and date of collection of all canine samples for the amplification of the rabies viral nucleoprotein (*N*) gene.

**Table 1:** Geographical origin and time of collection of the 19 dog samples used for amplification of the rabies virus nucleoprotein gene

Numbering of dog samples	Numbering of rabies virus isolates	Geographical origin of dog samples	Status of dogs	Time of sample collection
LY19	FJ001	Longyan	Seemingly healthy dogs	03/2007
LY36	FJ002	Longyan	Seemingly healthy dogs	03/2007
LY31	FJ003	Longyan	Seemingly healthy dogs	04/2007
QZ27	FJ004	Quanzhou	Seemingly healthy dogs	03/2007
QZ 31	FJ005	Quanzhou	Seemingly healthy dogs	03/2007
QZ 196	FJ006	Quanzhou	Seemingly healthy dogs	03/2007
QZ 260	FJ007	Quanzhou	Seemingly healthy dogs	03/2007
SM9	FJ008	Sanming	Rabies suspected dogs	11/2008
SM10	FJ009	Sanming	Rabies suspected dogs	11/2008
SM11	FJ010	Sanming	Rabies suspected dogs	01/2009
SM12	FJ011	Sanming	Rabies suspected dogs	01/2009
LY47	FJ012	Longyan	Seemingly healthy dogs	04/2007
LY48	FJ013	Longyan	Seemingly healthy dogs	03/2007
LY55	FJ014	Longyan	Seemingly healthy dogs	04/2007
SM2	FJ015	Sanming	Seemingly healthy dogs	11/2008
SM5	FJ016	Sanming	Seemingly healthy dogs	11/2008
SM14	FJ017	Sanming	Rabies suspected dogs	02/2009
SM15	FJ018	Sanming	Rabies suspected dogs	02/2009
SM16	FJ019	Sanming	Rabies suspected dogs	02/2009

### Detection of rabies virus antigen in dog saliva

Saliva specimens were collected from both rabid and seemingly healthy dogs housed in nine randomly selected villages from each city. For saliva sampling, each dog was housed in a cage, and sterile Q-tips/cotton balls were placed in mouth until Q-tips/cotton balls were thoroughly soaked by the saliva. Saliva specimens were kept in cold storage, immediately transferred to laboratory, pretreated by centrifugation and stored at −20 °C for subsequent tests. The rabies virus antigen was detected in saliva specimens using an enzyme-linked immunosorbent assay (ELISA) kit (Wuhan Institute of Biological Products; Wuhan, China) following the manufacturer’s protocol. In this study, a rabid dog was defined as a dog that actively attacked humans and animals and presented sympathetic hyperfunction, which showed manifestations of salivation, manic agitation and disturbance of consciousness.

### Amplification, cloning, sequencing and phylogenetic analysis of *N* gene in dog brain

A total of eight rabid dogs were sacrificed, and 81 seeming healthy (asymptomatic) dogs were euthanized. Canine brain tissues were collected and stored at −70 °C for the subsequent experiments. Total RNA was extracted from canine brain specimens using a Trizol reagent (Invitrogen; Carlsbad, CA, USA) according to the manufacturer’s instructions. *N* gene was amplified from the brain specimens using a nested reverse transcription polymerase chain reaction (RT-PCR) assay [7] with the primers described in Table 2 [8], while primers N1/N2 and N3/N4 were used for detection of virus nucleic acid, and primers N1/N5 and N6/N2 were used for the amplification of full-length *N* gene (Table 2).

**Table 2:** Primers used for nested RT-PCR amplification of the rabies virus nucleoprotein gene

Primer	Sequence (5′-3′)	Direction	Positions in genome	Paired primer	Product size (bp)
N1	ACAGACAGCGTCASATTGCAAAGC	Upstream	29–50	N2	1511
N2	TCGGATTGACGAAGATCTTGCTC	Downstream	1517–1539	N1	1511
N3	TTTGAGACTGCTCCTTTT	Upstream	587–605	N4	443
N4	CCCATATAGCATCCTAC	Downstream	1013–1029	N3	443
N5	CAGTCTCYTCNGCCATCT	Downstream	1570–1587	N1	1559
N6	ATGTAACACCTCTACAATGG	Upstream	55–74	N2	1485

PCR products were purified with the PCR product extraction kit (Omega Bio-Tek, Inc., Norcross, GA, USA), and cloned into the pMD18-T vector with a TA cloning kit (TaKaRa; Dalian, China) following the manufacturers’ instructions. Transformed clones were identified by restriction enzyme digestion and subjected to DNA sequencing. To increase the accuracy, we selected three clones of each fragment for sequencing. The sequence of the three clones that exhibited 100% homology was identified as the target sequence. Sequences were then aligned and compared with the *N* gene sequences of the reference strains obtained from GenBank using the software Bioedit version 7.0.1 (DNASTAR, Inc.; Madison, WI, USA) and MEGA version 4.0.

### Data analysis

All data were entered into Microsoft Excel 2007 (Microsoft, Inc.; Redmond, WA, USA), and all statistical analyses were performed using the statistical software SPSS version 13.0 (SPSS, Inc.; Chicago, IL, USA). Differences of proportions were tested for statistical significance using chi-square test, and a *P* value <0.05 was considered statistically significant.

## Results

### Epidemiological profile of human rabies cases

**Fig. 2:**
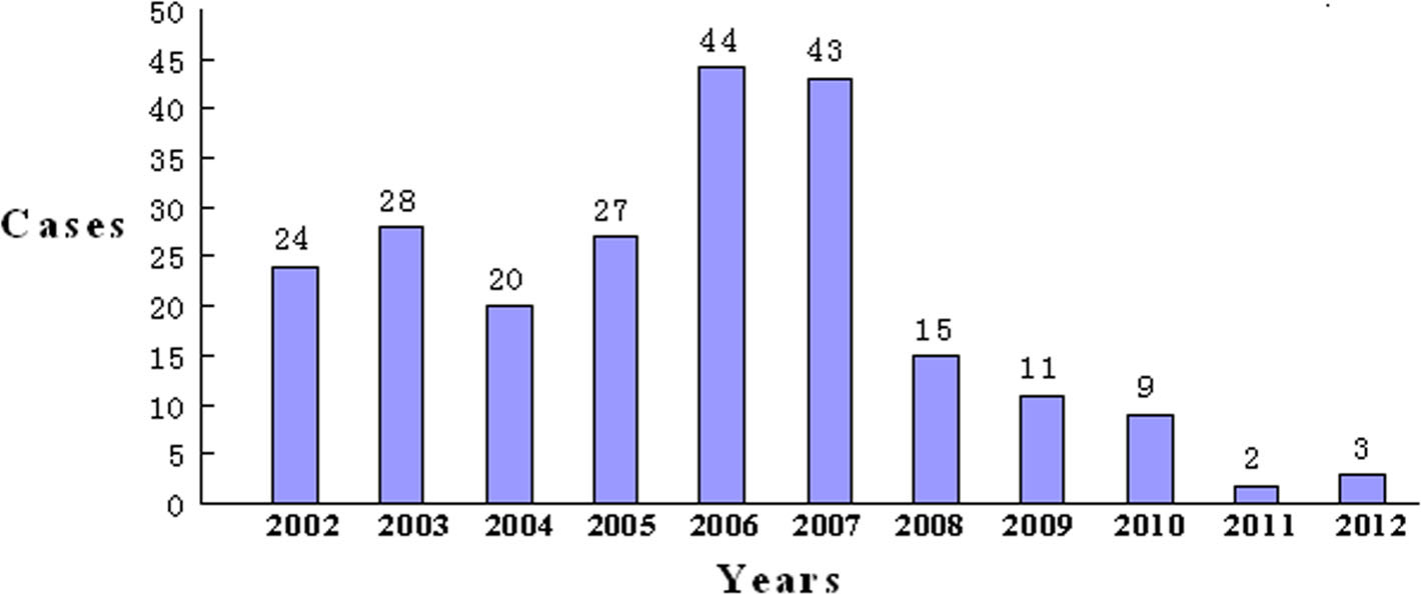
Annual rabies human cases reported in Fujian Province from 2002 to 2012

During the period from 2002 to 2012, a total of 226 human rabies cases were reported in Fujian Province, Southeast China. The cases included 147 men and 79 women, and had a mean age of 34.6 years (range, 1 to 88 years). The professions included farmers (138 cases), students (29 cases), workers (22 cases), preschool-aged children (14 cases), housekeepers (13 cases), and unknown (10 cases). The human cases were distributed across Fujian Province, including Quanzhou (85 cases), Longyan (67 cases), Sanming (47 Cases), Nanping (11 cases), Ningde (10 cases), Xiamen (3 cases), Fuzhou (1 case), Pingtan (1 case) and Putian (1 case), and 88.1% cases were reported in the three cities of Quanzhou, Longyan and Sanming. The annual number of human rabies cases is shown in Fig. 2.

### Rabies virus antigen in canine saliva

**Fig. 3:**
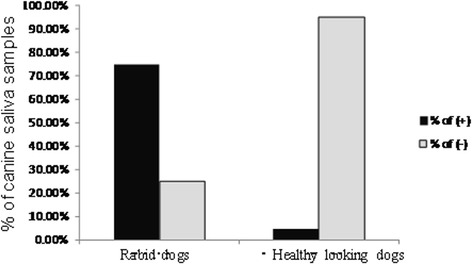
Detection of rabies virus antigens in rabid and seemingly healthy dogs

To investigate the incidence of rabies virus antigen in canine populations, a total of eight rabid dogs and 3492 seemingly healthy dogs were randomly selected from 27 villages of Quanzhou, Longyan and Sanming cities, of nine villages in each city, and dog saliva samples were collected. There were six of eight rabid dogs (75%) with detectable rabies viral antigen, and surprisingly, 165 of 3492 seemingly healthy dogs (4.7%) were positive for rabies virus antigens in saliva samples, suggesting that a considerable proportion of asymptomatic seemingly healthy dogs carries rabies virus (Fig. 3). There was a statistical significance in the incidence of rabies viral antigen between rabid and seemingly healthy dogs (*P* < 0.01).

### *N* gene fragments in canine brain specimens

Nested RT-PCR assay detected a 443 bp viral gene fragment in the brain specimens from seven of eight rabid dogs (87.5%) and 12 of 81 seemingly healthy dogs (14.8%), and then, all fragments were amplified with primers N1/N2 and N3/N4. Subsequently, the full-length *N* gene (1485 bp) was obtained from these positive specimens with primers N1/N5 and N6/N2. The amplicons were cloned into the pMD18-T vector, and subjected to DNA sequencing (GenBank accession numbers: FJ561726-FJ561732, FJ866827-FJ866831 and FJ866834-FJ866836).

### Phylogenetic analysis of full-length *N* genes

**Fig. 4:**
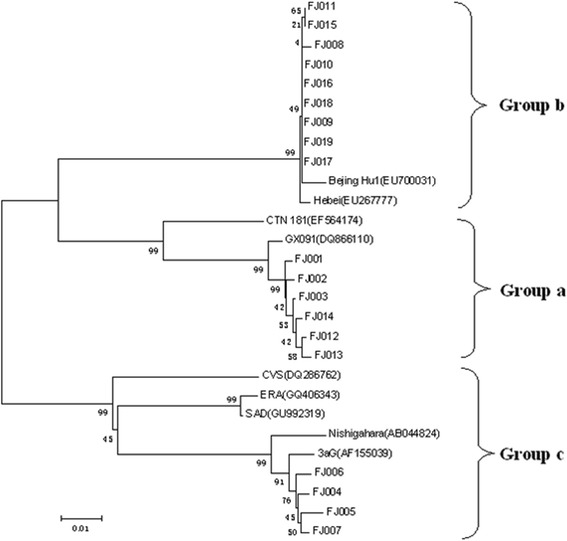
Phylogenetic analysis of amino acid sequences for rabies virus nucleoprotein. FJ001 to FJ019 rabies virus isolates were identified in this study, while the other sequences were downloaded from GenBank as reference sequences. Three genetically clustered groups were generated

Putative amino acid sequences of the 19 nucleoproteins were compared with the reference *N* gene sequence available in GenBank. Based on sequence alignment and phylogenetic tree analysis, these 19 *N* genes were characterized as the genotype I rabies virus, although three distinct clusters were found to be geographic region-specific (Groups A to C) (Fig. 4). Sequencing analyses revealed 99.7% to 100% intra-group homology of *N* genes, and 86.4% to 89.3% inter-group homology at a nucleotide level, and 98.9% to 100% intra-group homology and 95.3% to 98.4% inter-group homology at an amino acid level, respectively.

## Discussion

Analysis of the 1960–2014 notifiable surveillance data showed that rabies is still a serious public health concern in China, with a total of 120,913 human rabies cases reported in mainland China, including 19.8% cases observed during the period from 2004 through 2014 [9]. The highest incidence was recorded in 1981 (0.71/100,000), and in 2007 (0.25/100,000), and 59% of total cases were reported from June to November, including 11.0% of total cases observed in August. In addition, human rabies cases were reported in all provinces of in China, and the eastern and southern regions were more seriously affected relative to other regions [9]. Fujian Province is located in Southeast China, and is a high-risk environment for rabies [10]. The notifiable surveillance data showed 1894 human rabies cases during 1980–1989, 165 human cases during 1990–2000 [11], 206 human cases in 2000–2007 [12], and 123 human cases from 2006 to 2010 [5]. In 2013, only two human rabies cases and two deaths were observed in Fujian Province [13]. These data demonstrates an overall steady decline of human rabies cases in Fujian Province, Southeast China since 1980.

Rabies virus has a single-stranded, antisense, nonsegmented RNA virus genome (approximately 12 kb) encoding five viral proteins, including glycoprotein (G), nucleoprotein (N), phosphoprotein (P), matrix protein (M) and polymerase (L) [14]. *N* gene is highly conserved within the same subtype, and variations in this region can be used for virus subtyping and genotyping [15]. Nucleoprotein can protect viral nucleic acid from being damaged by host cell nuclease [16], and is one of the major protective antigens that induce cell-mediated immunity against rabies virus infection [17]. Molecular characterization of rabies virus *N* genes has been performed in some provinces of China [18–20]; however, the genotypes of dog rabies viruses remain unclear in Fujian Province, Southeast China to date.

This study is the first survey on rabies incidence in domestic dogs in Fujian Province, Southeast China. Saliva specimens, which were collected from rabid and seemingly healthy dogs in 27 villages of Quanzhou, Longyan and Sanming cities, were detected for rabies virus antigen by an ELISA assay, and 4.7% of seemingly healthy dogs were positive for rabies virus antigens. Then, dogs with ELISA-positive saliva were sacrificed and brain specimens were sampled for the subsequent nested RT-PCR assay. Surprisingly, nested RT-PCR assay detected a 443 bp viral *N* gene fragment in the brain specimens of 14.8% (12/81) of seemingly healthy dogs. The results suggest that there are a considerable proportion of false negatives in preliminary screening by ELISA. Indeed, the reliability, reproducibility and diagnostic value of rabies virus antigen detection remain in debate in canine saliva since the complicated components in saliva specimens may affect ELISA assays, and no standardized kits are available in market until now [21–23]. In addition, virus release occurs at limited intervals in infected dogs, and the difference in sampling time-points may partially contribute to great variations in rabies virus detection among different geographical regions, even inconsistent with local epidemiological conditions.

Sequencing analysis of the 19 full-length *N* genes revealed over 80% nucleotide and amino acid homology between rabies viral isolates form Fujian Province and other strains globally. Our findings demonstrate that seemingly healthy dogs may serve as carriers of rabies virus. Although the virus samples were captured from only three cities of the province, a considerable proportion of infected dogs inhabited in the communities without any obvious symptoms for a period of time, which is inconsistent with previous reports [24–26]. Nested RT-PCR assay established in this study was found to be more sensitive in detection of rabies in seemingly healthy dogs. The mechanism for infected dogs without illness onset is still unclear; however, seemingly healthy dogs have important values in public health and cannot be neglected.

Canine vaccination has been shown to be the most effective approach for the prevention of human rabies, and it is theoretically possible to eliminate cases of the deadly rabies virus in people worldwide through mass canine vaccination [27]. However, the majority of dogs in the developing world are not given vaccination, such as India, China, and Pakistan, which challenges the control efforts and global rabies elimination program [27]. Currently, the most widely used veterinary rabies vaccines include inactivated, attenuated and genetically engineered vaccines in the world [28, 29]. In China, only veterinary inactivated and attenuated rabies vaccines are available [30]. In this study, most of the dogs were free-ranging dogs in rural areas, which did not receive vaccination. It is therefore suggested that, on one hand, rabies virus vaccine inoculation for domestic animals like dogs and cats should be popularized for rabies prevention and control; on other hand, if a dog has no history of definite vaccination, medical personnel should give a standard treatment to dog-bitten outpatients, and vaccinate them according to recommended vaccination protocols, regardless of health status.

To date, there have been seven genotypes of rabies virus characterized [31]. Based on the sequence of the rabies virus *N* gene, six clearly distinct genotypes were distinguished according to their percentage of amino acid similarity, including the vaccinal and classical rabies viruses of genotype 1, genotypes 2 (Lagos bat virus) and 3 (Mokola virus), genotypes 4 (Duvenhage virus) and 5 (EBL1) and genotype 6 represented by European bat lyssaviruses (EBL) serotype 2 (EBLV-2) [32]. In addition, genotype 7 (Australian bat lyssavirus, ABLV) was isolated from Australian bats [33]. Currently, the majority of the rabies viruses isolated from human cases were characterized as genotype 1 [30]. In the present study, sequencing analysis revealed that the 19 full-length rabies virus *N* genes belonged to genotype I. According to their geographical origins, three distinct clusters (groups A, B, and C) were originated exclusively from western, northern and southern Fujian, respectively, which is in agreement with previous studies [34–36]. Phylogenetic analysis showed that the viral isolates in Group A shared the highest homology with a Guangxi isolate GX091, while viral isolates in Group B shared the highest homology with a Beijing isolate (Beijing Hu1). Notably, the viral isolates in Group C shared the highest homology with a vaccine strain 3aG and Nishigahara, a Japanese strain of veterinary vaccine. As a southeast coastal province of China, the complexity of rabies virus distribution in Fujian may reflect its geographic location with rapid economic development and frequent international communications. However, the detailed routes for rabies virus transmission cannot be determined among different cities to date, since limited molecular evidence is available in Fujian and neighboring regions.

The present study has several limitations. First, ELISA-positive saliva samples were not confirmed by RT-PCR. Our nested RT-PCR, which was employed to detect viral *N* gene in the brain specimens of dogs with ELISA-positive saliva samples, showed the low sensitivity (14.8%) of ELISA for the detection of rabies virus antigens in canine saliva specimens. As described above, ELISA detection is affected by multiple factors. Fortunately, the phylogenetic analysis is not affected, since the full-length N gene was amplified from PCR-positive samples. Second, we did not observe the dogs with ELISA-positive saliva samples for a period of time to see the eventual development of rabies or not. Third, we did not detect *G* gene in the canine samples. Currently, the rabies virus vaccine strains are CTN, 3aG, PV and Flury in Fujian province, and veterinary vaccine strains are ERA and SAD. Since G protein is the major inducer of protective immunity against rabies [37], we cannot link our findings with the rabies vaccination in Fujian Province.

## Conclusions

This is the first description pertaining to the epidemiological characteristics of human rabies cases and characterization of the rabies virus *N* gene in dogs in Fujian Province, Southeast China. Our findings may provide valuable knowledge for the development of strategies targeting the prevention and control of rabies. Further extensive surveillance is required to track virus evolution, and infection status in dogs and other carrier species.

## Abbreviations

CDC: Center for Disease Control and Prevention
ELISA: Enzyme-linked immunosorbent assay
G protein: Glycoprotein
L protein: Polymerase
M protein: Matrix protein
N protein: Nucleoprotein protein
P protein: Phosphoprotein
RT-PCR: Reverse transcription polymerase chain reaction
